# A mechanical rotation chair provides superior diagnostics of benign paroxysmal positional vertigo

**DOI:** 10.3389/fneur.2023.1040701

**Published:** 2023-01-27

**Authors:** Mathias Winther Bech, Alexander Torp Staffe, Dan Dupont Hougaard

**Affiliations:** ^1^Department of Otorhinolaryngology, Head and Neck Surgery and Audiology, Balance and Dizziness Centre, Aalborg University Hospital, Aalborg, Denmark; ^2^Department of Clinical Medicine, Aalborg University, Aalborg, Denmark

**Keywords:** vertigo, benign paroxysmal positional vertigo, mechanical rotation chair, repositioning chair, TRV chair, BPPV, diagnostics

## Abstract

**Background:**

Benign paroxysmal positional vertigo (BPPV) is the most common vestibular disease. Both therapeutic and diagnostic benefits with mechanical rotation chairs (MRCs) for management of BPPV have been reported. No previous studies have compared diagnostics in MRCs to traditional diagnostics on an examination bed.

**Objective:**

To investigate the agreement between BPPV diagnostics performed with an MRC and traditional diagnostics on an examination bed. Secondary objectives were to (1) examine if the two test modalities differ in diagnostic properties when diagnosing largely untreated patients referred from general practitioners (uncomplicated BPPV) compared to patients referred from private ENTs (complicated BPPV) and (2) examine whether impaired participant cooperation during Manual Diagnostics (MDs) alters agreement, sensitivity and specificity.

**Method:**

Prospective randomized clinical trial in which patients with a case history of BPPV were recruited by referrals from general practitioners, otorhinolaryngologists and other hospital departments in the Northern Region of Denmark. Participants underwent diagnostic examinations twice: once by traditional MDs on an examination bed and once with an MRC. Initial examiner and order of test modality were randomized. Examiners were blinded to each other's findings.

**Results:**

When testing the ability to diagnose BPPV, agreement between the two test modalities, was 0.83, Cohen's kappa 0.66. When comparing MD diagnostics to MRC diagnostics (set as gold standard diagnostics following test result interpretation), values for MDs were: sensitivity 71%, specificity 98%, Negative Predictive Value 73%, and Positive Predictive Value 97%. Agreement regarding BPPV subtype classification was found to be 0.71, and Cohen's kappa 0.58. Agreement when isolating the diagnosis to posterior canalolithiasis (p-CAN) was 0.89, Cohen's kappa 0.78.

**Conclusion:**

Diagnostics, aided by an MRC, are more sensitive than traditional manual BPPV diagnostics. The overall agreement level between test modalities was found to be weak to moderate. When isolating diagnostics to p-CAN, the level of agreement increased to “moderate-strong.” Results also showed higher agreement between test modalities and a significantly higher negative predictive value for MDs when examining patients referred directly from General Practitioners following no- or a single treatment attempt. The diagnostic properties of MDs improved in patients with a higher degree of cooperation.

## 1. Introduction

Benign paroxysmal positional vertigo (BPPV) is the most common vestibular disease, accounting for 17–42% of patients with vertiginous symptoms (1). The disease is caused by the dislodgement of otoconia from the utricular macula into one or several of the semicircular canals (SCCs) within the inner ear.

BPPV is classified according to (1) the anatomical location of otoliths dislodged from the utricle and (2) the manner in which otoliths disturb endolymphatic flow (2). An accurate classification therefore includes the following additional information regarding location, laterality, and subtype, respectively: posterior, lateral, and/or anterior SCC(s), left and/or right SCC(s), and canalolithiasis (CAN) and/or cupulolithiasis (CUP) (3, 4).

Despite the fact that highly effective treatment options exist, and spontaneous resolution rates range from 27 to 50%, BPPV may still significantly impair general health and quality-of-life (5). Approximately 86% of patients with BPPV experience interruptions in daily activities and lost days at work due to BPPV. Elderly patients with BPPV are at greater risk of falls, depression, and impaired daily function (6). The adverse quality-of-life impacts reported amongst BPPV patients are aggravated by long delays in placing the correct diagnostics and initiation of the appropriate treatment. The time from the onset of symptoms until the correct diagnostic tests are carried out, has been reported to be in the order of months, due to a combination of patient- and doctor delays (7, 8). Additionally, with a lifetime prevalence estimated to be 2.4%, BPPV constitutes a substantial healthcare burden (9). The American Academy of Otolaryngology—Head and Neck Surgery Foundation (AAO-HNS) estimates the costs associated with a diagnosis of BPPV alone to approach two billion dollars annually (4). BPPV-associated costs and delays in diagnostics are both exacerbated by the frequent use of unnecessary diagnostic tests, as reports show that MRIs, CT-scans, various pharmaceutical treatments, and electrocardiograms are frequently prescribed prior to placing a correct diagnosis of BPPV (10). Therefore, improvements in BPPV diagnostics and subsequent targeted treatment of BPPV-patients may significantly increase quality-of-life measures amongst BPPV patients and reduce individual as well as societal costs.

Traditionally, BPPV is diagnosed by a clinician following standardized diagnostic tests where the patient is placed in a number of specified head positions to provoke both an objective characteristic positional nystagmus and a subjective concomitant feeling of vertigo (4). The Committee for Classification of Vestibular Disorders of the Bárány Society and the AAO-HNS have both formulated diagnostic criteria for BPPV that rely on (1) A characteristic case history and (2) specific positional nystagmus patterns during diagnostic testing. While the AAO-HNS criteria also list concomitant subjective vertigo in association with the objective positional nystagmus as a diagnostic criterion, The Barany criteria rely on patient history and objective findings alone (3).

The diagnostic accuracy of these tests is subject to both examiner- and patient-dependent variables that may affect both intra- and inter-examiner reliability, e.g., the speed of head movements and the specific angle of the head position during testing (11). One study estimated the positive predictive value (PPV) of the manual Dix-Hallpike (DH) test to be 83% and the negative predictive value (NPV) to be 52% (12). Another study estimated the sensitivity and specificity of the DH test to be 79 and 75%, respectively (13), suggesting that efforts to improve diagnostic testing may be beneficial in cases where symptomatology is highly suggestive of BPPV, but traditional examinations fail to place the correct diagnosis.

Several devices that may aid BPPV diagnostics have been developed: (1) *optical Frenzel goggles* that provides magnification of the eyes of the patient and reduce visual fixation, (2) *videonystagmography (VNG)* that yields similar benefits as optical Frenzel goggles, but further improve nystagmus assessments as the eyes of the patient are enlarged on a TV-screen for a more detailed view and replayed for further investigation or second opinion (4). VNG goggles also enable quantification and characterization of nystagmus with analysis of parameters such as direction (horizontal, vertical, rotational), beats per minute, and average slow phase velocity measurements, (3) *mechanical rotation chairs* ensure standardization of diagnostic test procedures, potentially providing increased intra- and inter-examiner reliability, the utilization of MRCs reduces patient-dependent factors that may affect the quality of diagnostic tests (e.g., neck stiffness and reduced mobility, reduced patient cooperation etc.) to a minimum. Lastly, MRCs facilitate treatment options not possible with traditional repositioning maneuvers carried out on an examination bed, (4) the combination of *MRCs* and *VNG goggles* adds and utilizes the benefits of both approaches.

A number of biaxial MRCs have been developed, including the Epley Omniax Rotator^®^ (Vesticon©, Portland, USA), the TRV^®^ MRC (Interacoustics©, Middelfart, Denmark) and the Rotundum positioning chair (Balcare, Küsnacht, Switzerland). These companies all supplement their respective MRCs with VNG usage. With the biaxial MRC used in this study, the angles of the yaw axis are fixed in predefined positions and the examiner controls the speed of head-movements, thus facilitating diagnostic and therapeutic procedures in patients who would otherwise be unable to cooperate. The examiner-dependent speed of head movements during the positional testing does probably account for some degree of inter- and intra-examiner variability, as the speed of head movements may vary between examiners as well as between tests carried out by the same examiner. The MRC, which is used in this study, allows 360-degree rotations in the roll axis and 360-degree rotations with 45-degree predetermined intervals in the yaw axis, minimizing patient-dependent diagnostic factors such as head movement velocity and head position angles, and allows more standardized test and treatment protocols. The concomitant use of infrared video goggles enables usage of software features such as pupil auto tracking and slow-phase velocity measurements that may assist the examiner in interpreting the positional nystagmus observed (14). It has recently been hypothesized that the predefined and highly reproducible movements with MRCs, combined with VNG goggles, provide superior diagnostics of BPPV (15).

Though a number of studies have reported therapeutic and diagnostic benefits of using MRCs for management of BPPV (14, 16–18), no studies have directly compared the agreement of diagnostic procedures done with an MRC in combination with infrared video goggles to the diagnostic procedures done with manual diagnostic (MD) tests as carried out in a primary care setting.

Therefore, the primary objective of this study was to investigate the agreement between BPPV diagnostics performed with an MRC and MDs on an examination bed. Secondary objectives of this study were to (1) examine if the test modalities differ in sensitivity and specificity when diagnosing uncomplicated BPPV cases [referred from general practitioners (GPs)] compared to complicated BPPV cases [referred to tertiary care from private otorhinolaryngologists (ENTs)] and (2) examine if impaired patient cooperation during MDs alters agreement, sensitivity and specificity.

## 2. Methods and materials

### 2.1. Participants

Between September 1st, 2021, and November 30th, 2021, adult patients were referred to the tertiary Balance and Dizziness Center at the Department of Otolaryngology, Head and Neck Surgery and Audiology, Aalborg University Hospital, Aalborg, Denmark. Patients were referred from GPs, private ENTs and other hospital departments in The North Denmark Region (total population size of ~600.000).

**Figure 1:**
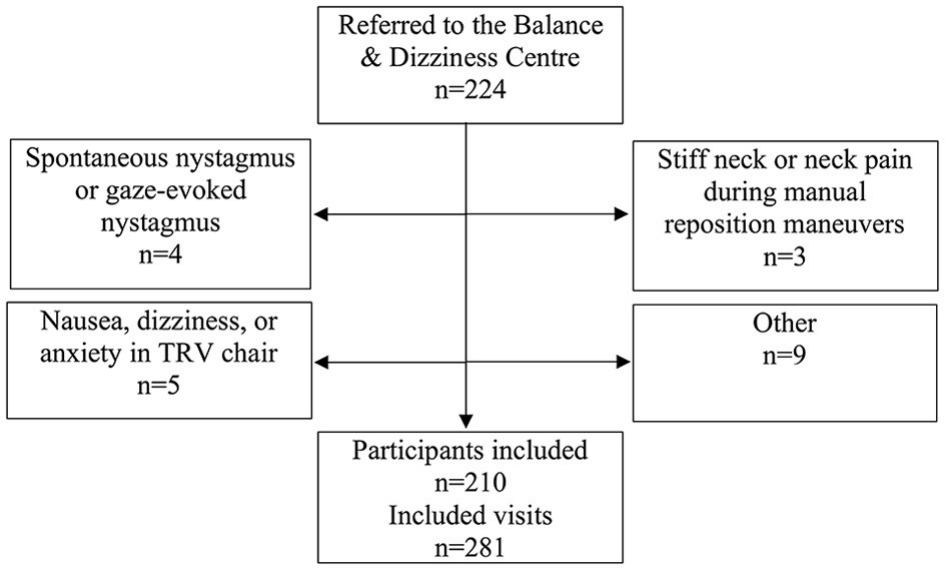
Trial profile. Please note that participants could be excluded at one visit but included at a separate follow-up visit if the reason for exclusion was reversible.

All participants included in the study had a case history of paroxysmal positional vertigo and/or a clinical presumption of BPPV at the time of referral. 224 participants were referred, 21 visits were excluded (see Figure 1), and therefore a total of 210 participants were included. All participants included in the study, who were diagnosed with BPPV, were invited to an in-house follow-up 2–3 weeks after initial diagnostics and treatment. At follow-up visits, prior to follow-up history taking and prior to re-examinations, participants were allowed to be included in the study again, if a new consent form was obtained. There were no additional requirements for re-inclusion in the study as these patients still fulfilled the initial inclusion criteria of a typical case history of BPPV. Furthermore, at follow-up, the examiners did not know if previous treatment(s) had been successful or unsuccessful, and therefore they did not know if the patient did or did not have any positional nystagmus. This approach resulted in a total of 281 inclusions with 210 unique patients.

Participants referred from private ENTs were characterized by long lasting and persistent symptoms despite several (or at least more than one) manual BPPV treatments on an examination bed prior to referral and were thereby categorized as complicated cases of BPPV. Conversely, participants referred from GPs or other hospital departments were characterized by a shorter symptomatic period and no more than one traditional manual treatment attempt. Patients were referred directly from GPs and were included as uncomplicated cases, if they had undergone up to one treatment session. GPs were instructed not to go forward with CRPs, as patients would be assessed and subsequently treated within a few days of referral throughout the duration of this study. Therefore, almost all patients referred directly from GPs had no previous BPPV treatment *prior* to referral. Similarly, patients with suspected BPPV would be referred directly from other hospital departments without previous treatment attempts.

### 2.2. Materials

All patients underwent screening tests for spontaneous- and gaze-evoked nystagmus as well as a rotation test including VOR suppression. Patients with abnormal findings on screening tests were excluded from the study and submitted to subsequent exams post-study if relevant. Since all patients included presented with a typical BPPV case history and had to display nystagmus patterns compatible with BPPV in order to receive the BPPV diagnosis, extensive neurological examinations were not routinely performed.

**Figure 2:**
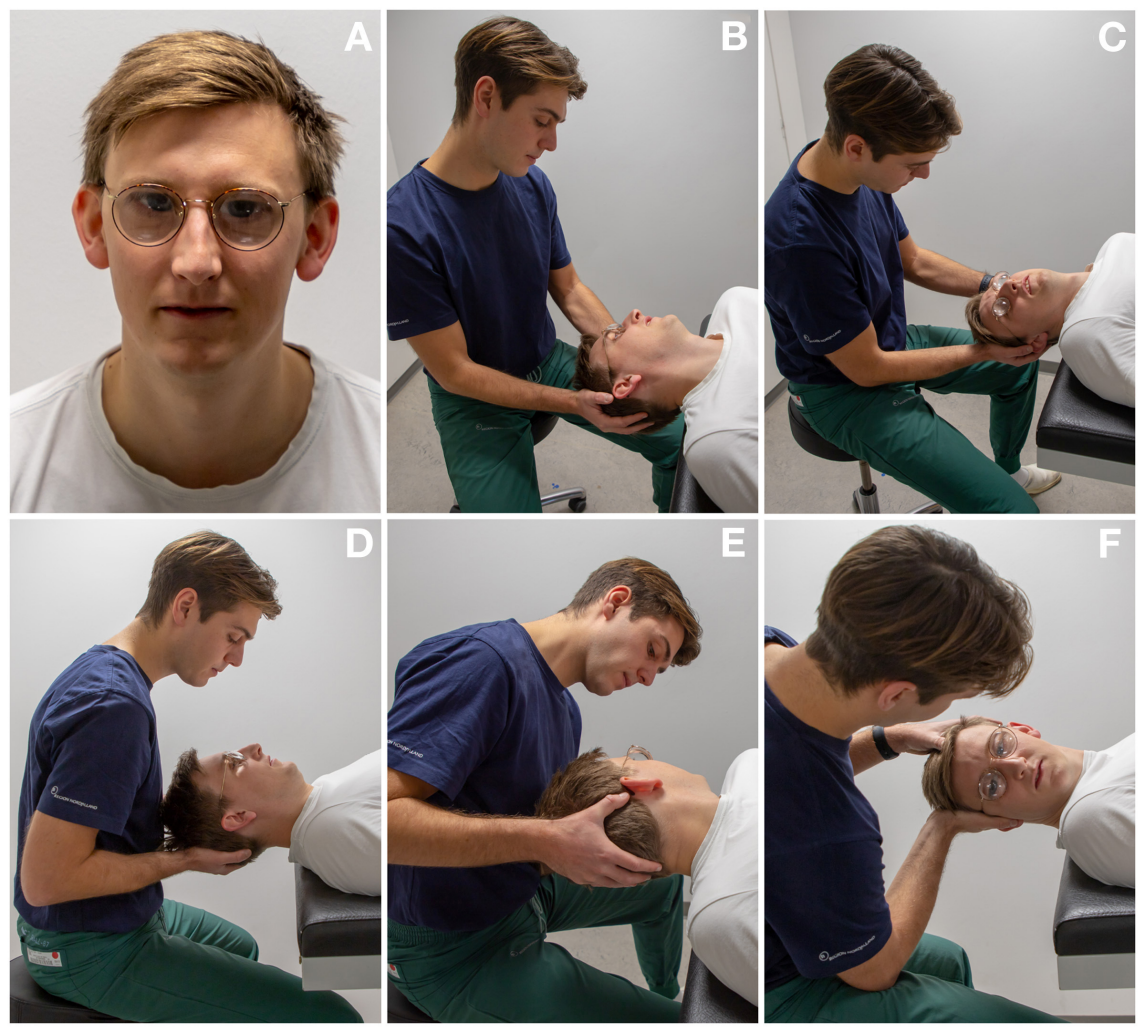
Diagnostics as performed on an examination bed: **(A)** optical Frenzel goggles were used for manual diagnostics. **(B, C)** Left and right Dix-Hallpike tests: the participant's head is turned 45° to each corresponding side and the participant's neck is extended 20°. **(D)** Supine position: the participant's head is lightly flexed. **(E, F)** Left and right Supine Roll test: from the supine position, the participant's head is turned 90° to each corresponding side.

Manual diagnostic tests were performed with the aid of optical Frenzel goggles specifically designed to magnify the eyes of the participant and to reduce possible fixation. The diagnostic tests were meticulously conducted in the following order for every patient regardless of findings: Left DH test, right DH test, supine position, left Supine Roll Test (SRT) and right SRT, see Figure 2. Bilateral DH tests were done in order to diagnose ipsilateral posterior and/or contralateral anterior BPPV, while SRTs were used to diagnose lateral BPPV. Individual sequential positions during DH tests and SRTs were maintained for 60 s.

**Figure 3:**
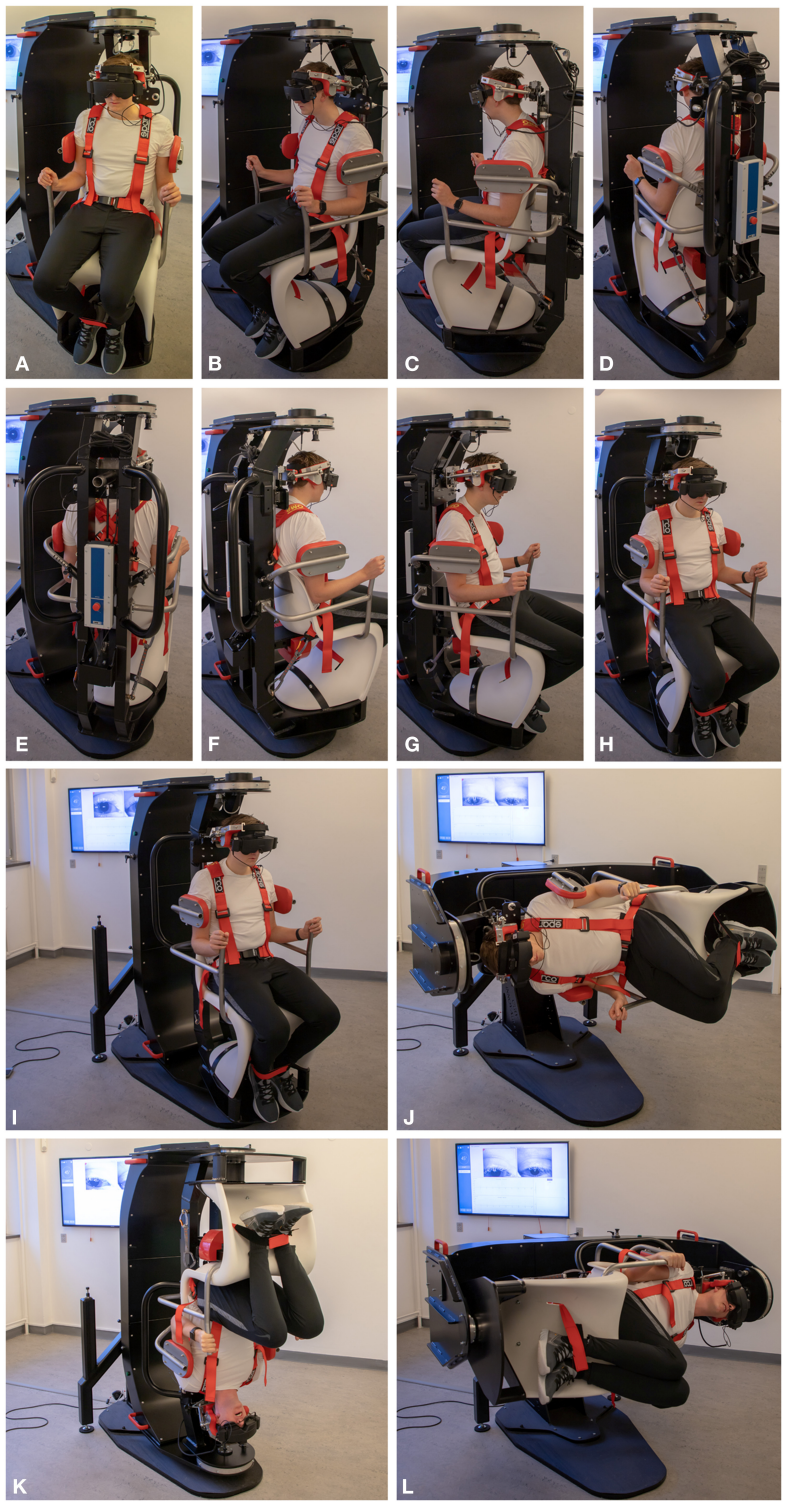
Degrees of freedom in the TRV mechanical rotation chair: **(A–H)** 360° rotations with 45° predetermined intervals in the yaw axis. **(I–L)** 360° rotations in the roll axis.

**Figure 4:**
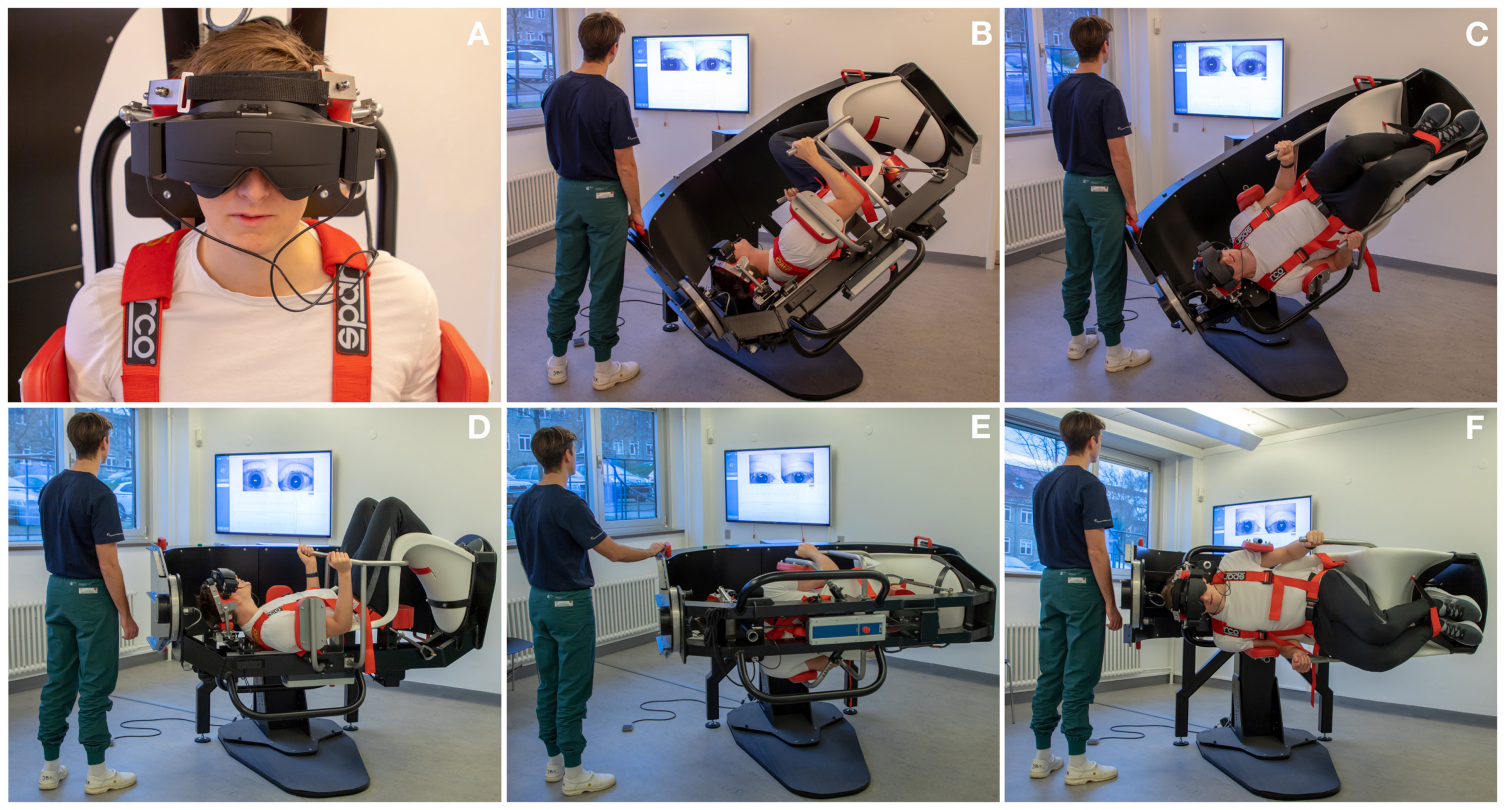
Diagnostics as performed in the mechanical rotation chair: **(A)** Videonystamography goggles were used for diagnostics in the mechanical rotation chair. **(B, C)** Left and right Dix-Hallpike tests: the participant is turned 45° in the yaw axis to one side and 120° backwards in the roll axis with the affected ear pointed downwards. **(D)** Supine position: the participant is turned 90° backwards in the roll axis. **(E, F)** Left and right Supine Roll test: from the supine position, the participant is turned 90° in the yaw axis to each side.

Participants were diagnosed using corresponding tests with the TRV^®^ MRC (Interacoustics©, Middelfart, Denmark). Participants were fitted with VNG goggles (VF405^®^, Interacoustics©, Middelfart, Denmark), see Figure 3. The accompanying software (OtoAccess version 3.0.0.1) enabled quantification and characterization of nystagmus with analysis of parameters such as direction (horizontal, vertical, and rotational) and average slow phase velocities. Diagnostic tests with the MRC were conducted in the same order and with equal duration of time as with the MDs, see Figure 4.

BPPV was diagnosed and subcategorized according to the Bárány criteria (3), see Table 1. The term non-posterior BPPV was used in this study to group all BPPV subtypes and -locations other than posterior CAN (the by far most common subtype and location), into one single group. This allows comparisons between the diagnosis of the most prevalent BPPV subtype and -location and the rather heterogenous group of less common BPPV subtype and/or -locations.

**Table 1:** BPPV subclassification according to nystagmus characteristics observed with the Dix-Hallpike and the supine roll tests.

**Semicircular canal**	**Canalolithiasis**	**Cupulolithiasis**
Posterior SCC	Ipsilateral Dix-Hallpike: upbeat nystagmus with a torsional component. Short latency. Crescendo-decrescendo pattern. Duration <1 min	Like canalolithiasis. Without latency. Nystagmus persistent > 1 min
Lateral SCC	Supine roll test: geotropic nystagmus. Nystagmus and vertigo more intense on affected side. Short latency. May present with crescendo-decrescendo pattern. Duration <1 min	Supine roll test: apogeotropic nystagmus. Nystagmus and vertigo more intense on nonaffected side. No latency. Nystagmus persistent > 1 min
Anterior SCC	Contralateral Dix-Hallpike: Downbeat nystagmus with or without a torsional component. Duration <1 min	Like canalolithiasis. Nystagmus persistent > 1 min.

SCC, semicircular canal.

Based upon etiology, patients diagnosed with BPPV were classified as primary or secondary BPPV. Secondary BPPV included patients with a recent head trauma (time wise correlation with the onset of vertiginous symptoms) or patients with concurrent inner ear disease(s) that predisposes to the development of BPPV. Patients with no evident etiology were classified as primary or idiopathic BPPV.

### 2.3. Design

Prospective Randomized Clinical Trial. Participants underwent diagnostic testing twice: once by means of MDs on an examination bed and once by means of an MRC. All tests were performed by the same two examiners. Initial examiner and type of initial diagnostic examination was randomized. Block randomizations were used with blocks of 12. Examiners were blinded to each other's diagnostic test results. The two diagnostic tests were separated by a minimum of 15 min (start of initial examination until the initiation of the following examination) and always performed by the same two examiners sequentially and independently. All participants diagnosed with BPPV were allowed to be included more than once, if they were scheduled for a follow-up examination. Therefore, if participants re-consented at follow-up examinations, the additional set of diagnostics for that participant was included in the study. As a direct result of this, a higher number of total inclusions than individual participants are seen. Subsequently, all patients diagnosed with BPPV were offered targeted CRP treatment with an MRC (not part of the study).

People included in the study were also asked to rate the severity of their symptoms from zero to three with the following descriptions in mind: none: 0, mild: 1, moderate: 2, severe: 3. Immediately following diagnostic tests, study participants were instructed to rate their symptoms during diagnostic testing with the MRC and the MD separately.

Patient cooperation during MDs was rated and divided into the following three groups: (1) adequate cooperation, (2) impaired, yet acceptable cooperation, and (3) unacceptable cooperation. Adequate cooperation was defined as: (a) bilateral DH tests in which the patient's head was *rotated 45 degrees* to the corresponding side and moved from a sitting to a lying position with *20-degrees head extension* in one *continuous fluent motion in *<*2 s*, with a total examination time of *60 s* in both DH positions, and (b) bilateral SRTs in which the patient's head was *rotated 90-degrees* to the corresponding side from the supine position in one continuous fluent motion *lasting* <*2 s*, and with a total examination time of *60 s* in both SRT positions. Impaired, yet acceptable cooperation was defined as: (a) DH tests in which the patient's head was *rotated at least 30 degrees* to the corresponding side and moved from a sitting to a lying position with *at least 10-degrees head extension*, and (b) SRTs in which the patient's head was *rotated at least 2/3* of the targeted *90 degrees* to the corresponding side from the supine position with an examination time for all positional tests of *60 s*. Examinations, that failed to fulfill the listed group 1 and 2 criteria, were classified as “unable to cooperate,” and were excluded from this study and instead offered diagnostics in an MRC. Reasons for reduced cooperation (groups 2 and 3) included: neck stiffness, neck pain, obesity, anxiety, and severe dizziness.

### 2.4. Statistical analysis

Baseline characteristics were described by descriptive statistics. The agreement between the two test modalities was analyzed by percentages agreement and Cohen's kappa. For Cohens kappa, the two test modalities were considered the raters. In this study, as proposed by Warrens (19), Cohen's kappa is used and interpretated as the proportion of agreement when corrected for chance. McHugh suggested the Kappa result be interpreted as follows: values 0–0.20 as indicating no agreement and 0.21–0.40 as minimal, 0.41–0.60 as weak, 0.61–0.80 as moderate, 0.81–0.90 as strong, and 0.9–1.00 as almost perfect agreement (20). Results are presented with 95% confidence intervals.

The agreement between the two test modalities was analyzed with regards to the ability of the individual test modality to facilitate a diagnosis of BPPV overall (termed “BPPV and no BPPV”) as well as the agreement between test modalities regarding BPPV characteristics in terms of (1) laterality (left/right), (2) location [SCC(s) being affected], and (3) subtype (CAN and/or CUP)—altogether termed “agreement on laterality, location and subtype.

Sensitivity, specificity, NPVs and PPVs were calculated to compare the two test modalities. The most sensitive test modality overall was chosen as “gold standard” in subsequent calculations. The test modality, that provoked the largest amount of BPPV-characteristic positional nystagmus, cf. the Bárány criteria for BPPV, was defined as the most sensitive test modality.

Study data were collected and managed using REDCap electronic data capture tools hosted at a secure server with log function at The North Denmark Region (21, 22). REDCap^®^ (Research Electronic Data Capture) is a secure, web-based software platform designed to support data capture for research studies. Stata/MP 17.0 software was selected for the processing and analysis of data, and the processing and analyses were completed with support from a certified biostatistician.

## 4. Results

In this study, a total of 281 inclusions were made. Baseline characteristics are based upon the 281 inclusions and not exclusively the 210 individual participants. Baseline characteristics are shown in Table 2. Females accounted for 63.7% of inclusions and the mean age was 62.2 years, ranging from 22 to 96 years of age. Participants were referred from private ENTs (34.5%), GPs (61.9%), and other hospital departments (3.6%). Primary BPPV (89.7%) was more common than secondary BPPV (10.3%).

**Table 2:** Baseline characteristics.

**Characteristics**		**Total**
Age (years), mean (±SD) [range]		62.2 (15.9) [22–96]
**Sex**, ***n*** **(%)**		
Male inclusions		102 (36.3)
Female inclusions		179 (63.7)
**Duration (min) between test modalities, mean (±SD) [range]**		23.8 (7.2) [15–52]
**Participants**, ***n***		210
Included one time		154
Included two times		44
Included three times		10
Included four times		1
Included five times		1
**Inclusions**, ***n*** **(%)**		281
– With BPPV, *n* (%)		160 (56.9)
***Private otorhinolaryngologists***		97 (34.5)
– *With BPPV*		76 (78.4)
– *With p-CAN*		49 (64.5)
– *With non-p-CAN*		27 (35.5)
– *With primary BPPV*		66 (86.8)
– *With secondary BPPV*		10 (13.2)
***General practitioners***		174 (61.9)
– *With BPPV*		79 (45.4)
– *With p-CAN*		52 (65.8)
– *With non-p-CAN*		27 (34.2)
– *With primary BPPV*		72 (91.1)
– *With secondary BPPV*		7 (8.9)
**Primary BPPV in total**, ***n*** **(%)**		142 (50.5)
**Secondary BPPV in total**, ***n*** **(%)**		18 (11.2)
– *Head trauma*		14 (77.8)
– *Vestibular neuritis*		3 (16.7)
– *Morbus Menière*		1 (5.6)
**BPPV characteristics**	**MD**	**MRC**
– **BPPV laterality**, ***n***		
*Left*	38	58
*Right*	46	52
*Bilateral*	30	47
– **BPPV SCC Location**, ***n***		
*Posterior SCC*	78	81
*Lateral SCC*	13	30
*Anterior SCC*	7	10
*Multi-canal*	16	36
– **BPPV Subtype**, ***n***		
*Canalolithiasis*	89	93
*Cupulolithiasis*	16	44
*Both*	9	20

p-CAN, posterior canalolithiasis. BPPV characteristics between inclusions referred from private otorhinolaryngologists and general practitioners. MD, manual diagnostics; MRC, mechanical rotation chair.

In 121 (43.1%) inclusions, the two test modalities both rejected the BPPV diagnosis. In 111 (39.5%) of inclusions, we were able to place a diagnosis of BPPV with both test modalities. With the remaining 49 (17.4%) of cases, diagnostics with the MRC allowed a diagnosis of BPPV in 46 out of 49 (93.9%) cases with BPPV while MD allowed a diagnosis of BPPV in three out of 49 (6.1%) cases, see Table 3. Therefore, the MRC was assumed to be the most sensitive test modality and was categorized as “gold standard” in subsequent sensitivity and specificity calculations.

**Table 3:** Diagnostics of BPPV.

**Mechanical rotation chair**	**MDs**		**Total**
	***BPPV***	***No BPPV***	
*BPPV*	111	46	157
*No BPPV*	3	121	124
Total	114	167	281

MRC diagnostics enabled the examiners to place a BPPV diagnosis in 46 out of 49 participants where the two test modalities disagreed in BPPV diagnostics.

Agreement between the two modalities, when testing the ability to detect BPPV, was found to be 0.83 (95% CI [0.78; 0.87]), Cohen's kappa: 0.66 (95% CI [0.58; 0.74]). When comparing MDs to diagnostics done with the MRC (gold standard), sensitivity was 71%, specificity was 98%, NPV was 73%, and PPV was 97% (see Table 4). When consensus on laterality, location and subtype, e.g., right-sided horizontal CAN, was a requirement for agreement, the agreement percentage was found to be 0.71 (95% CI [0.66; 0.77]), Cohen's kappa: 0.58 (95% CI [0.51; 0.65]).

**Table 4:** Overall agreement, sensitivity, specificity and predictive values with selected BPPV properties.

	***n*, (%)**	**Agreement**		**MDs compared to a mechanical rotation chair**			
		**Percentage agreement** **[CI 95%]**	**Cohen's kappa** **[CI 95%]**	**Sensitivity** **(%)**	**Specificity** **(%)**	**NPV** **(%)**	**PPV** **(%)**
**All inclusions**	281 (100%)						
BPPV or no BPPV		0.83 [0.78; 0.87]	0.66 [0.58; 0.74]	71	98	73	97
Agreement on laterality, location and subtype		0.71 [0.66; 0.77]	0.58**^#^** [0.51; 0.65]				
Agreement on SCC affection with p-CAN		0.89 [0.85; 0.92]	0.78**^#^** [0.71; 0.85]				
**Inclusions with BPPV but no p-CAN**	56 (19.9%)						
BPPV or no BPPV		0.39 [0.26; 0.52]	−0.07 [−0.17; 0.03]	41	^*^	^*^	92
Agreement on laterality, location and subtype		0.23 [0.12; 0.35]	0.17 [0.06; 0.28]				

p-CAN, posterior canalolithiasis; SCC, semicircular canal ^*^Cannot be estimated.

# Please note that Cohen's kappa in agreement on SCC affection with p-CAN is significantly higher than for overall agreement on laterality, location and subtype.

Diagnostics isolated to single SCC affection with posterior CAN are described in Table 5. Agreement was calculated to be 0.89 (95% CI [0.85; 0.92]), Cohen's kappa: 0.78 (95% CI [0.71; 0.85]). Non-posterior BPPV was diagnosed in 56 (19.9%) inclusions. Agreement on laterality, location and subtype between the two test modalities in the 56 inclusions was found to be 0.23 (95% CI [0.12; 0.35]), Cohen's kappa: 0.17 (95% CI [0.06; 0.28]). When analyzing the ability to diagnose non-posterior BPPV, the agreement was 0.39 (95% CI [0.26; 0.52]), Cohen's kappa: −0.07 (95% CI [−0.17; 0.03]).

**Table 5:** BPPV diagnostics isolated to the BPPV subtype of posterior canalolithiasis.

**MDs**	**Mechanical rotation chair**				**Total**
	**No** **p-CAN**	**Right** **p-CAN**	**Left** **p-CAN**	**Bilat** **p-CAN**	
No p-CAN	177	6	9	1	193
Right p-CAN	1	34	3	5	43
Left p-CAN	2	0	31	4	37
Bilat p-CAN	0	0	1	7	8
Total	180	40	44	17	281

p-CAN, posterior canalolithiasis; Bilat, bilateral.

The 56 inclusions were asked to rate the severity of their symptoms (none: 0, mild: 1, moderate: 2, severe: 3). The means for the MRC and the MDs were found to be 0.91 and 0.52, respectively, reflecting that ratings for the severity of symptoms significantly increased when using the MRC (*P* < 0.05).

Inclusions were divided into subgroups according to: (1) the referring physician, (2) the degree of participant cooperation during MDs, and (3) initial diagnostic test modality (following randomization), refer to Table 6. Agreement, in percentage, between test modalities in patients who underwent MDs first, was 0.83 (95% CI [0.77; 0.89]), Cohen's kappa 0.66 (95% CI [0.54; 0.78]), sensitivity 72%, specificity 96%, NPV 74%, and PPV 95%. The percentage agreement, between test modalities patients who underwent MRC diagnostics first, was 0.82 (95% CI [0.76; 0.89]), Cohen's kappa 0.66 (95% CI [0.54; 0.78]), sensitivity 69%, specificity 100%, NPV 71%, and PPV 100%.

**Table 6:** Overall agreement, sensitivity, specificity and predictive values with selected subgroups.

	***n*, (%)**	**Agreement (BPPV or no BPPV)**		**MDs compared to a mechanical rotation chair**			
		**Percentage agreement** **[CI 95%]**	**Cohen's kappa** **[CI 95%]**	**Sensitivity** **(%)**	**Specificity** **(%)**	**NPV** **(%)**	**PPV** **(%)**
**Referred from**							
Private ENTs	97 (34.5)	0.79 [0.71; 0.88]	0.54 [0.38; 0.71]	75	96	53	98
General practitioners	174 (61.9)	0.83 [0.78; 0.89]	0.65 [0.54; 0.76]	65	98	78	96
**Cooperation at MDs**							
Adequate cooperation	224 (79.7)	0.85 [0.80; 0.89]	0.70 [0.61; 0.79]	73	98	76	98
Impaired, yet acceptable cooperation	53 (18.9)	0.75 [0.64; 0.87]	0.52 [0.32; 0.73]	66	95	58	96
**First test modality**							
Mechanical rotation chair	136 (48.4)	0.82 [0.76; 0.89]	0.66 [0.54; 0.78]	69	100	71	100
MDs	145 (51.6)	0.83 [0.77; 0.89]	0.66 [0.54; 0.78]	72	96	74	95

Inclusions are subdivided into groups according to referrals, degree of cooperation during MDs, and initial test modality. NPV, negative predictive value; PPV, positive predictive value; ENTs, otorhinolaryngologists; the differences in NPV related to referrals and degree of cooperation during MDs.

## 5. Discussion

This study is one of few studies who have attempted to quantify the accuracy of positional tests in BPPV diagnostics and, to our knowledge, is the first study to compare MDs on an examination bed to diagnostics aided by an MRC combined with VNG goggles.

In accordance with the first Bárány criteria, all participants included in this study presented with a typical case history of BPPV (3). Additionally, a diagnosis of BPPV was only placed if clearly defined objective criteria (specific positional nystagmus patterns) were met. By applying strict and well-defined diagnostic criteria, the most sensitive test modality may be determined and classified as the superior diagnostic tool.

The results of this study demonstrate that an MRC is a considerably more sensitive, and thereby a more accurate, diagnostic tool for placing a diagnosis of BPPV compared to MD testing on an examination bed. This finding has also been suggested in a recent study that compared the treatment efficacy of an MRC to traditional manual repositioning maneuvers (15).

Our results show that the agreement between MRC diagnostics and MDs regarding the overall detection of BPPV, is moderate when interpreting Cohen's kappa as suggested by McHugh (20). In 46 out of the 49 cases (93.9%), where the two test modalities disagreed on the diagnostics of BPPV, positional testing with the MRC was able to place a diagnosis of BPPV.

Our findings reconfirm that MD on an examination bed is a useful diagnostic tool in cases where BPPV is the suspected diagnosis. This is supported by the calculated values for sensitivity (71%), specificity (98%), NPV (73%), and PPV (97%). However, a sensitivity and NPV of 71 and 73%, respectively, indicate that a negative test result may require further testing if the case history is highly suggestive of BPPV. In such cases, patients may benefit from a subsequent reexamination in an MRC. NPVs should be interpreted with caution and solely as values for cohorts which include patients with a case history compatible with BPPV as well as a concomitant clinical suspicion of BPPV raised by either a GP or an ENT-specialist, and therefore not as values applicable to the general population.

A positive test, however, should be considered an unambiguous indication of BPPV, and manual treatment maneuvers should be initiated. A study by Schuricht and Hougaard (15) has shown that treatment of posterior BPPV with the same MRC and traditional manual repositioning maneuvers were equally sufficient. However, another study by Tan et al. (18) found that treatment with the same MRC was superior one and 3 months following initial treatment. No significant difference was detected 6 months after the initial treatment.

The most common subtype and location of BPPV, posterior CAN, seems more suited for MDs than other BPPV locations and/or -subtypes. The results of this study show a moderate to strong level of agreement (20) when testing the ability to diagnose posterior CAN specifically. When analyzing the 56 cases of non-posterior BPPV, agreement on BPPV detected is interpreted as none. This strengthens the recommendation that MRC aided diagnostics should be considered if a patient is diagnosed with non-posterior BPPV and exact classification in terms of localization, subtype, and laterality proves challenging.

Existing literature has shown that patients with complicated BPPV are diagnosed with non-posterior BPPV more frequently when examined in an MRC, suggesting that less common types of BPPV on occasion may be overlooked by MDs (14, 17). This study found that when agreement on laterality, location and subtype, e.g., right-sided horizontal CAN, is a requirement for agreement between modalities, agreement between test modalities is characterized as weak when interpreting Cohen's kappa as suggested by McHugh (20). Since correct and accurate BPPV diagnostics, including determination of BPPV laterality, subtype, location, is a prerequisite for adequate treatment, our findings suggest that patients with complicated BPPV may benefit from examination and treatment with an MRC—for diagnostic as well as therapeutic purposes.

A secondary objective of this current study was to investigate whether agreement between test modalities differs in participants suffering from complicated BPPV compared to uncomplicated cases of BPPV. This study subdivided the population into a group of participants with complicated BPPV and a group of participants with uncomplicated BPPV. The uncomplicated group consisted of participants referred from GPs. The complicated group was defined as participants referred from private ENTs due to complicated BPPV. Our results show higher agreement and a significantly higher NPV in the uncomplicated group while a higher sensitivity for BPPV was calculated in the complicated group. These results suggest that MDs are best utilized when examining uncomplicated cases of BPPV. Measurements of sensitivity and NPV in the uncomplicated group should be interpreted with caution due to a high number of participants without BPPV (55.9%) in this group.

Our study also aimed to investigate if the degree of participant cooperation during MDs influenced the agreement between test modalities. Not surprisingly, results show (4, 11), that agreement, sensitivity, specificity, NPV and PPV are higher when the examiner noted “adequate cooperation,” compared to cases with impaired, but acceptable cooperation. Our findings suggest that an MRC should be considered in patients where MDs are compromised due to a low degree of patient cooperation.

### 5.1. Existing literature

Though no previous studies have directly analyzed the agreement between MDs and diagnostics aided by an MRC, a few studies concerned with the evaluation and improvement of BPPV diagnostics have been published. Andera et al. (23) compared the standard DH test to a modified DH test intended to enhance cupular displacement during the positional nystagmus testing. They found that the modified DH test appeared to improve test sensitivity, worsen symptoms during testing and increase the duration of nystagmus. In accordance with this present study, the findings published by Andera et al. indicate that even rather subtle adjustments to positional provocative BPPV tests may significantly improve their sensitivity.

Hanley et al. (12) monitored the use of DH tests performed by a number of GP's in Northwest Ireland. They calculated the PPV of a positive DH test to be 83.3% while the NPV was 52%, thereby producing estimates for predictive values lower than those calculated in this study (PPV = 97.4%, NPV = 72.5%). Clear differences in methodological approaches may account for these differences, as the validity of the initial BPPV diagnosis was controlled through telephone contact with the participating doctor 1 month after each participant's initial consultation, constituting a feeble basis for a “gold standard.” Another study, conducted by Cohen (24), monitored positional nystagmus with electrodes rather than through real-time nystagmus observations. Cohen found similar results regarding sensitivity (79.3%) and PPV (95.8%) but a much lower specificity (75%) and NPV (33.3%). The study compared the DH test against an unspecified “gold standard,” while this present study compared the use of the DH test and the SRT against analog tests performed with an MRC. Lopez-Escámez et al. (25) defined their “gold standard,” as the selection of the same diagnosis by three independent clinicians and estimated the sensitivity of the DH test at 82% and the specificity at 71%.

Apart from the abovementioned studies, very few recent studies concerned with the accuracy of BPPV tests exist. The basis for direct comparison between findings in previous publications and the agreement, sensitivity and specificity data in this current study is, therefore, rather weak. In existing literature, the DH test is widely considered the “gold standard,” in the diagnosis of posterior and anterior canal BPPV while the SRT is the most commonly utilized tool for diagnosing lateral BPPV. Because of this, investigating the properties and utility of positional tests for BPPV faces a significant challenge: Positional tests cannot truly be 100% sensitive for BPPV due to the intermittent nature of the condition, however, the diagnosis depends on these tests (13). Though specific BPPV biomarkers and references for imaging findings have been studied, none are currently deemed appropriate for clinical use (26, 27). The absence of an alternative external gold standard to positional BPPV tests limits the amount of sensitivity and specificity data currently published (4).

### 5.2. Methodological considerations

The distribution of BPPV subtypes reported in this study deviates from previously reported frequency rates. In this study, 104 (65%) participants were diagnosed with posterior BPPV, while 56 (35%) participants were diagnosed with non-posterior BPPV. As non-posterior BPPV has been estimated to account for 10–20% of BPPV cases (4, 28, 29), the distribution of BPPV subtypes found in this study shows a remarkably higher proportion of non-posterior BPPV. A number of studies concerned with the utilization of MRCs in the treatment of BPPV participants have reported findings similar to this study. Pedersen et al. (14), Schuricht and Hougaard (15), and West et al. (17) all reported a tendency toward larger proportions of non-posterior BPPV when using MRCs for diagnostics. Because of this, it has been speculated that the frequency of non-posterior BPPV has been underestimated in studies that do not apply MRCs or VNG for diagnostics. They estimate that the true proportion of non-posterior BPPV is closer to 30%, which is closely aligned with the findings in this study (15). Schuricht and Hougaard (15) and West et al. (17) hypothesized that at least some of the participants presenting with non-posterior BPPV would have been diagnosed insufficiently without the application of MRCs. Findings in this study strongly support this hypothesis, as the sensitivity of MD tests compared to diagnostics aided by an MRC was estimated at just 41% for non-posterior BPPV. Part of the increase in non-posterior BPPV cases may be explained by the fact that ~35.5% of the tests conducted in this study were performed with participants referred from private ENTs with complicated BPPV, since non-posterior BPPV is known to be more treatment (30). However, participants referred directly from GPs displayed a similar distribution (34.2%) in this study.

A well-established feature associated with CAN BPPV is that the nystagmus typically fatigues with repeated provocative tests (31). Since participants included in this study were to be examined twice on the same visit, the study design was outlined in a manner intended to account for the fatigability of BPPV-induced positional nystagmus. This was done by separating the initiation of the respective examinations by a minimum of 15 min and by randomization of the order in which they were performed. Imai et al. (32) studied the recovery of positional nystagmus after BPPV fatigue and found that the effect of BPPV fatigue disappeared within 30 min. In this present study, following an average duration of 23.8 min between the initiation of the two test modalities, values for sensitivity, specificity, PPV, and NPV were nearly identical in the group subject to MDs first (Sen: 72%, Spe: 96%, NPV: 74%, and PPV: 95%) compared to the group examined with the MRC first (Sen: 69%, Spe: 100%, NPV: 71%, and PPV: 100%). Potential BPPV fatigability, therefore, did not seem to affect the results of this study. The findings of this study indicate that the fatiguability of BPPV-related positional nystagmus is clinically insignificant, when applying a minimum of 15 min between the initiation of two consecutive positional tests.

The present study compares MDs using optical Frenzel goggles to diagnostics performed in an MRC using VNG goggles. This design prevents us from estimating the extent to which the MRC *and* the inclusion of VNG, respectively, increase sensitivity. As the objective of this study was to compare contemporary BPPV diagnostics, as performed in primary care and emergency departments, to the diagnostics performed at highly specialized tertiary care units, this was a conscious decision on the authors' part. Future studies that directly compare the diagnostic properties of manual tests aided by VNG goggles with manual tests aided by optical Frenzel goggles are strongly encouraged as well as additional studies directly comparing BPPV diagnostics aided by optical Frenzel goggles on both an examination bed and with an MRC. The latter would clarify if the MRC alone also increases diagnostic accuracy.

Martens et al. (33) hypothesized that the use of VNG goggles for the detection of positional nystagmus leads to BPPV overdiagnosis. They studied positional nystagmus in 78 healthy adults and detected positional nystagmus in 88% of the subjects. The 95th percentile of the maximum average slow-phase velocity (aSPV) for each subject was found to be ~5° and 6.5° per second in the lateral and vertical planes, respectively. We hypothesize that the high prevalence of positional nystagmus amongst healthy subjects observed by Martens et al. may lead to overdiagnosis of BPPV in clinics where concomitant positional vertigo, provoked by positional testing, is not included in the diagnostic criteria for BPPV. Our study did not apply specific cut-off limits for the velocities of the observed positional nystagmus, but all participants were diagnosed by trained medical personnel with an adequate clinical understanding of BPPV and all included participants had a classical case history compatible with BPPV. Application of specific aSPV cut-off values with positional nystagmus together with the addition of a supplementary prerequisite of a clear association between the objective positional nystagmus and the concomitant subjective vertigo during positional testing would most likely prevent or limit the degree of BPPV overdiagnosis, if these parameters were to be included as part of the diagnostic criteria.

## 6. Conclusion

The results of this study show that diagnostics aided by an MRC are more accurate than traditional manual BPPV diagnostics. However, when isolating diagnostics to posterior CAN, the level of agreement increases. Results also show higher agreement between test modalities and a significantly higher NPV for MDs when examining patients referred from GPs mostly without previous treatment attempts (uncomplicated BPPV patients). The diagnostic properties of MDs decline in cases with impaired participant cooperation.

In summary, traditional MDs on an examination bed is a useful first line diagnostic tool. For diagnostics purposes, an MRC may be considered under the following circumstances: (1) When the BPPV diagnosis is not unambiguous; especially in patients where symptomatology is highly suggestive of BPPV, but traditional positional tests fail to place the diagnosis, (2) cases with non-posterior BPPV; particularly if exact localization proves challenging, (3) Patients with persistent BPPV following more than one treatment session from an ENT specialist, (4) when MDs are compromised due to impaired or low degree of patient cooperation.

## Data availability statement

The raw data supporting the conclusions of this article will be made available by the authors, without undue reservation.

## Ethics statement

The studies involving human participants were reviewed and approved by the North Denmark Region Committee on Health Research Ethics, Denmark (approval number N20210038). The patients/participants provided their written informed consent to participate in this study. Written informed consent was obtained from the individual(s) for the publication of any potentially identifiable images or data included in this article.

## Author contributions

Concept and supervision: DH. Design, materials, analysis and/or interpretation, writing manuscript, and critical review: DH, MB, and AS. Data collection and/or processing and literature search: MB and AS. All authors contributed to the article and approved the submitted version.
